# Eating Quality and In Vitro Digestibility of Brown Rice Improved by Ascorbic Acid Treatments

**DOI:** 10.3390/foods12051043

**Published:** 2023-03-01

**Authors:** Qin Wei, Yubao Guo, Kang Tu, Xiuling Zhu, Dan Xie, Xinyu Liu

**Affiliations:** 1 School of Biological and Food Engineering, Anhui Polytechnic University, Wuhu 241000, China; 2 College of Food Science and Technology, Nanjing Agricultural University, Nanjing 210095, China

**Keywords:** brown rice, ascorbic acid, hydrothermal treatment, texture, crystallinity, in vitro digestion

## Abstract

The effects of ascorbic acid treatment alone and in combination with degreasing or hydrothermal treatment on eating quality and in vitro digestibility of brown rice were explored for improving poor mouthfeel and low digestibility, and the improvement mechanism was investigated. The results indicated that the texture of cooked brown rice was significantly improved by degreasing combined with ascorbic acid hydrothermal treatment; the hardness and chewiness decreased to the level of polished rice; the stickiness increased three times of the cooked untreated brown rice; and the sensory score and in vitro digestibility were significantly enhanced from 68.20 and 61.37% to 83.70 and 79.53%, respectively. In addition, the relative crystallinity and water contact angle of treated brown rice were respectively reduced from 32.74% and 113.39° to 22.55% and 64.93°, and normal temperature water uptake significantly increased. Scanning electron microscope showed that the separation of starch granules occurred inside cooked brown rice grain obviously. The improvement of eating quality and in vitro digestibility of brown rice is conducive to enhancing the consumers acceptance and human health.

## 1. Introduction

Almost half of the world’s population lives primarily on rice. It is frequently consumed in the form of milled rice by removing the bran layer from brown rice, which is also referred as polished rice [1]. The bran layer of brown rice contains a variety of health-promoting components, such as vitamins, minerals, functional antioxidants, bioactive ingredients, and also contains more proteins, lipids, and dietary fibers than endosperm [1,2,3]. Therefore, brown rice is more beneficial than polished rice. However, the bran layer may hinder water uptake and starch gelatinization of brown rice during cooking, resulting in low digestibility, undesirable chewiness, and hard texture of cooked brown rice, thus popular consumers have not widely accepted brown rice [2].

Several methods have been reported to decrease the cooking time of brown rice and enhance the mouthfeel and digestibility of cooked brown rice, such as partial milling, freeze-thaw treatment, pregelatinization, germination, enzymatic hydrolysis, and ultrasonic treatment [3,4,5,6,7]. Nevertheless, these treatments are still insufficient to overcome the disadvantages of unpleasant odors, poor appearance, undesirable texture, and mouthfeel for brown rice consumption [2]. Soaking is a basic method to promote water diffusion into rice grains. In addition, it also improves the palatability of cooked rice to a certain extent [8]. However, prolonged soaking at room temperature possibly provokes microbial contamination, thereby affecting the color, mouthfeel, and odor of the product. Increasing soaking temperature has been reported to facilitate water diffusion into the grains and starch gelatinization during cooking, leading to softer texture, and reducing microbial pollution [9]. Previous studies have shown that the pasting viscosity and starch digestibility of germinated brown rice decreased after hydrothermal treatment [10]. The medium-temperature hydrothermal treatment has a significant effect on the hardness and chewiness of brown rice, but the improvement in stickiness is not satisfactory, and the effect of medium-temperature hydrothermal treatment on the digestibility of brown rice remains unknown.

The lipid content of brown rice is about 1.6~2.8%, which is higher than polished rice, mainly distributed in the rice bran layer [11]. The rancidity of the lipids commonly leads to a sour odor, making brown rice more intolerant of storage and unacceptable to the majority of consumers [12,13]. The bran layer of brown rice is rich in protein, which is a high-quality plant protein beneficial to human health [11]. Higher protein and lipid contents, owing to retained bran layers of brown rice, were described to hinder water uptake and extend the cooking time [12]. It was also reported that the texture of oats was effectively improved and the antioxidant properties of oat flour were enhanced by degreasing, hydrothermal, and drying treatments [14]. Degreasing of brown rice has been shown to improve the permeability of rice bran layer and promote water uptake of rice grains, thus improving the texture. However, the effect of degreasing on the stickiness of cooked brown rice is limited, and its effect on starch digestibility of cooked brown rice is unclear.

Ascorbic acid is a small molecule that can easily penetrate into the gap between starch granules and is a cheap and safe food-grade additive [15]. Ascorbic acid has been used as a reducing agent in the storage of various fruits and vegetables [16,17,18]. A few studies have focused on improving the texture and pasting properties of aged rice and investigating the aging mechanisms of rice with ascorbic acid treatment before or after rice storage [19,20,21]. It was reported that reducing agent promoted aged rice to absorb water fully by the mechanism of cleaving disulfide bond into sulfhydryl groups to form a sticky paste, and the gelatinized paste layer thickened and softened the cooked rice [20]. It was reported that high-pressure freeze-thaw cycles and germination-parboiling treatments could decreased the hardness of brown rice and improved its digestibility [22]. Similarly, ascorbic acid was also found to significantly improve the stickiness of cooked aged rice to the target of cooked fresh rice by promoting the swelling and separation of starch granules in rice grain [23]. Up to now, studies on the effects of ascorbic acid on the eating quality and in vitro starch digestibility of brown rice have not been found.

The objective of this work was to investigate the effects of ascorbic acid treatment alone or in combination with degreasing and/or hydrothermal treatment on eating quality and in vitro digestibility of cooked brown rice, and provides an effective way to prepare the prefabricated brown rice for sale in the market and easier cooking by consumers. The changes in cooking quality, relative crystallinity, water droplet contact angle, normal temperature water uptake of treated brown rice, and microscopic morphologies inside the cooked treated brown rice were investigated to reveal the underlying reasons for the improvements of texture, sensory, and in vitro digestibility.

## 2. Materials and Methods

### 2.1. Materials and Reagents

Non-waxy japonica brown rice (BR) and polished rice (PR), “Nanjing 9108” cultivars, Oryza sativa L., were harvested in Xuyu County, Jiangsu Province, China, whose moisture content was 13.56 ± 0.72% and 13.12 ± 0.48% respectively. Ascorbic acid (≥99.0%) and absolute ethanol (≥99.5%) were purchased from Sino Pharm Chemical Reagent Co., Ltd. (Shanghai, China). Pepsin (250 U/mg protein), α-Amylase (250 U/g solid), pancreatin (8 × USP), and invertase (300 U·mg^−1^ solid) were purchased from Sigma-Aldrich Ltd. (St Louis, MO, USA). Amyloglucosidase (3260 U/mL), the total starch assay kit and the glucose assay kit were all obtained from Megazyme International Ireland Ltd. (Wicklow, Ireland).

### 2.2. Brown Rice Treatments

Brown rice was degreased according to the reference method [24] to obtain degreased brown rice (BR-D). Brown rice (BR, 40 g) was degreased with four volumes of absolute ethanol based on the weight of brown rice by magnetic stirring at 25 °C for 1 h. After filtering by filter paper, the degreased brown rice grains were naturally volatilized for 24 h at normal temperature and then dried to its initial moisture content at 35 °C to obtain degreased brown rice (BR-D). Degreased brown rice contains 1.15 ± 0.09% lipids, whereas the material brown rice contains 1.91 ± 0.03% lipids. Brown rice (two portions) was soaked in 100 mmol/L ascorbic acid solution (pre-experiment for improvement effect) for 1 h at normal temperature [21], and then one portion was dried to initial moisture content at 35 °C to obtain ascorbic acid-treated brown rice (BR-A). The other one was immediately hydrothermal-treated at 80 °C for 30 min, and then dried to the initial moisture content at 35 °C to obtain ascorbic acid hydrothermal-treated brown rice (BR-AH). The same treatments were applied to degreased brown rice to respectively obtain degreasing combined with ascorbic acid-treated brown rice (BR-DA) and degreasing combined with ascorbic acid hydrothermal-treated brown rice (BR-DAH).

### 2.3. Rice Cooking and Texture Measurement

Rice sample of 8.0 g was washed twice and soaked in distilled water (7.2 mL) for 1 h in an aluminum cup covered with tinfoil and then cooked as described by Ohno et al. [20]. After steaming for 20 min and holding temperature for 20 min in an electric rice cooker (CFXB15-5M, Weiduofu Electric Industrial Co., Ltd., Guangzhou, China), the cooked rice was cooled down at 25 °C for 90 min. Texture Analyzer (TA. XTC, Bosin Tech, Shanghai, China) was used to measure the texture of the cooked rice using the reference method [21]. More than 20 cooked rice grains of each sample were measured. The measurement conditions were as follows: load cell, max. 5 kgf; plunger, 25 mm diameter; compression deformation, 90%; plunger and stage, aluminum; bite speed, 1 mm/s; sample temperature at measurement, 25 °C.

### 2.4. Sensory Evaluation of Cooked Rice

Sensory evaluation was carried out by the method with minor modifications [4,25,26]. In this research, the cooked rice samples were evaluated using descriptive analysis (Table S1). The sensory evaluation procedure was conducted in a sensory laboratory following national standard (GB/T 15682-2008). Ten semi-trained sensory panelists (5 Male and 5 Female, age range 20–30) were selected and trained to evaluate the cooked rice. Semi-trained sensory panelists refer to the national standard (GB/T 15682-2008) to select sensory evaluators with high sensory sensitivity through identification experiments. The sensory panelists were trained to understand the basic techniques and methods of sensory analysis and basic knowledge about products, so as to improve their ability to detect, identify, and describe sensory stimuli refer to ISO 8586-1 procedure. The training items include detection and recognition of tastes and odors, the use of scales, the development and use of descriptors, and training in evaluation of cooked rice samples. The number of participants in the training is 1.5 to 2 times the number of sensory panelists required, and all training trials should be conducted in a suitable environment. The candidate sensory panelists after training need to pass the examination and become real sensory panelists. For the description test of cooked rice samples, more than 80% accuracy should be achieved before they can become sensory panelists through the training. Before tasting each sample, the panelists were required to rinse their mouth thoroughly with purified water. Each cooked rice sample (20 g) was transferred to plastic cups and presented to the panelists using a random sample order. The panelists tasted one sample at a time in individual booths chew three times, and evaluated sensory attributes including odor, appearance, palatability, flavor, and cold texture described in Table S1.

### 2.5. In Vitro Digestion of Cooked Rice

Simulated in vitro salivary-gastrointestinal digestion of cooked rice was performed using a reference method with minor modifications [27]. Weigh 2 g rice grains, fully cook and cool it as a sample for standby. For saliva digestion, α-amylase solution (10 mg α-amylase dissolved in 10 mL 100 mmol/L sodium acetate buffer with pH 6.8) was added to the cooked rice sample, and the sample was shaken for 2 min at 37 °C. After saliva digestion, the gastrointestinal tract was digested according to the method reported by Tian et al. [27]. Then, simulated digestion supernatants (0.5 mL) were collected at different time points throughout small intestinal digestion processes (5, 10, 15, 20, 30, 60, 90, 120, 150, 180, 240 min) and immediately diluted with 2.5 mL of 95% ethanol to inactivate the enzymes. The glucose concentrations of the incubated mixtures were measured using a glucose analysis kit, and the starch content of brown rice was measured with the total starch assay kit.

A standard curve for starch digestion at different glucose concentrations was constructed, which followed Equation (1) [28].

C_t_ = C_∞_ (1 − e^−kt^) (1)

where C_∞_ is the estimated percentage of final starch digestion, C_t_ is the starch digestibility at t min, and k is the starch digestion rate coefficient. Then the area under the curve was calculated.

Equation (2) was provided to calculate the glycemic index (GI) [28].

GI = 39.71 + (0.549 × HI)(2)

where hydrolysis index (HI) was calculated by dividing the area under the curve of the sample by the corresponding area of white bread.

### 2.6. Cooking Quality of Rice

The glass plate-white nucleus method was used for the measurement of optimal cooking time (OCT) described by Mohapatra et al. [29]. Heating water uptake (HWU), volume expansion rate (VER), and solid loss (SL) were determined by the reference method [7]. Whole grains of 10 g were placed in boiling distilled water and kept boiling during test using a heating jacket (SKM, Hualu Electrothermal Instrument Company, Juancheng, Shandong, China). The cooking time was recorded from the time that the rice grains were put into the boiling water. After cooking for 15 min, 10 rice grains were taken out every one-minute interval, and the grains were pressed with two glass plates. The time that the 90% white cores of 10 rice grains disappeared plus 2 min was taken as the OCT of the tested sample. Rice grains (10 g) were soaked in distilled water of 100 mL in an aluminum box covered with aluminum foil and cooked in an electric cooker until the OCT. After cooking, cooked rice grains were drained and cooled to normal temperature (25 °C). The cooked rice grains were weighed and the HWU was calculated as the weight percentage of water uptake during cooking compared to material brown rice. The volumes of cooked and uncooked rice grains were measured, and the VER of the sample during cooking was calculated as the percentage of increased volume during cooking compared to material brown rice volume. The supernatant was collected in aluminum container and dried to constant weight at 105 °C. The SL of rice grains was calculated as the weight percentage of the solids in supernatant compared to material brown rice.

### 2.7. Relative Crystallinity of Rice

The X-ray diffractometer (D8-Advance, Bruker AXS Inc., Karlsruhe, Germany) was used to characterize the crystalline structure of the starch in treated brown rice. The scanning rate was 5.0°/min and the scanning range (2θ) was 5–40° [30]. Moreover, the relative crystallinity is calculated by dividing the area of the crystalline region by the sum of the areas of the crystalline and amorphous regions in X-ray diffractograms [30].

### 2.8. Water Droplet Contact Angle of Rice

The hydrophilicity of brown rice grain was characterized by water droplet contact angle with a contact angle meter (SL250, KINO Industry, Boston, MA, USA). A droplet of distilled water (1.5 μL) was dropped on the surface of rice grain and then the contact angle was obtained by sessile drop method [31]. Analysis was performed immediately after deposition of a single droplet of distilled water on the grain surface. Further, the dynamics of the droplet shape were recorded for 10 s by a video camera.

### 2.9. Normal Temperature Water Uptake of Rice

Normal temperature water uptake (NTWU) of rice grains was obtained by measuring weight difference before and after soaking [32]. Rice grains (8 g) were soaked in distilled water at a constant temperature of 25 °C for 1 h, then the surface water of rice grains was wiped off and weighed. The NTWU was calculated as the weight percentage of water absorbed and material brown rice (Equation (3)).

T_1_ = (M_i_ − M_0_)/M_0_ × 100% (3)

where T_1_ is the NTWU, %; M_i_ is the mass of brown rice after soaking, g; M_0_ is the mass of brown rice before soaking, g.

### 2.10. Microscopic Morphology Observation

The freeze-dried cooked rice grains were ground and then passed by a 105 μm sieve screen to obtain appropriate particles in size for observation. The scanning electron microscope (SEM, S-4800, Hitachi company, Tokyo, Japan) was used to scan and take photographs of the sample at an accelerating voltage of 5 kV.

### 2.11. Statistical Analysis

All data were determined in triplicate, unless otherwise indicated, and the results were expressed as mean ± standard deviation. The data were further processed through analysis of variance (ANOVA) by Duncan’s multiple range tests (*p* < 0.05). All statistical analyses and graphs were performed by using SPSS Software (Version 19.0.) and Origin 2022 software, respectively.

## 3. Results and Discussion

### 3.1. Effects of Ascorbic Acid Treatments on Texture of Cooked Brown Rice

The effects of ascorbic acid treatments on the texture of cooked brown rice were shown in Table 1. Cooked polished rice (PR) has lower hardness (H) and chewiness, and higher stickiness (S) and S/H ratio. Cooked untreated brown rice (BR) has the highest hardness and chewiness, and the lowest stickiness and S/H ratio, which was consistent with the poor mouthfeel and chewing difficulty of cooked brown rice, therefore it was not popular with most of the consumers. Brown rice treated with ascorbic acid alone (BR-A) presented a significant decrease in hardness and chewiness, as well as an increase in stickiness and S/H ratios. Degreasing combined with ascorbic acid treatment (BR-DA) did not further reduce the hardness and chewiness, but further enhanced the stickiness and S/H ratio. It is concluded that degreasing was helpful to increase the stickiness of cooked brown rice. Compared with ascorbic acid treatment alone, ascorbic acid hydrothermal treatment (BR-AH) not only significantly decreased the hardness and chewiness, but also increased the stickiness and S/H ratio, showing that the texture of cooked brown rice was further improved, the four indexes were still significantly different from polished rice in all four cases. Degreasing combined with ascorbic acid hydrothermal treatment (BR-DAH) not only markedly increased the stickiness and S/H ratio but also decreased the hardness and chewiness of cooked brown rice to the target of cooked polished rice, which were respectively 65.13% and 65.22% of the stickiness and S/H ratio of cooked polished rice, more than three times of the cooked untreated brown rice. The improving impact of degreasing combined with ascorbic acid hydrothermal treatment on the texture of cooked brown rice was very significant, also showing that degreasing was necessary to enhance the stickiness of brown rice.

It is the gelatinization degree of starch granules in rice grain that determines the texture of cooked rice [33]. The cooked untreated brown rice has high hardness and chewiness, which is consistent with the results reported by Huang et al. [34], which may be caused by insufficient water uptake and uneven water diffusion during cooking. Cooked brown rice with ascorbic acid alone or combined with degreasing or hydrothermal treatment decreased in hardness and chewiness, which may be attributed to the improvement of water uptake capacity of brown rice. It was reported that the bran layer outside brown rice grain was rich in proteins and lipids, which may hinder water uptake during soaking [35]. It can be seen that the stickiness of cooked brown rice is the most difficult to be improved among the four texture indexes listed in Table 1. In general, the more soluble solids dissolved, the greater the stickiness of cooked brown rice. Ascorbic acid, as a small molecule, has been found to increase soluble solids and thus enhance the stickiness of aged rice [21]. The partial removal of lipids from brown rice by absolute ethanol can reduce the preventing effect of lipids on water uptake, and benefit the water penetration and the solid leaching during ascorbic acid treatment, which is believed to improve the texture of cooked brown rice. After hydrothermal treatment, the increased tiny holes were observed on the bran layer of brown rice grain [24], which helps to increase water uptake channels, and ascorbic acid treatment was speculated to promote the separation of starch granules and form more water uptake channels inside brown rice grain, which is beneficial to solid leaching. Therefore, degreasing combined with ascorbic acid hydrothermal treatment can evidently increase the stickiness of cooked brown rice.

### 3.2. Effects of Ascorbic Acid Treatments on Sensory Scores of Cooked Brown Rice

**Figure 1:**
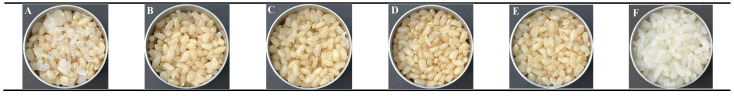
Effects of ascorbic acid treatments on appearance of cooked brown rice. The letters of (**A**–**F**) show the appearance of the cooked BR, BR-A, BR-DA, BR-AH, BR-DAH, and PR, respectively. BR, BR-A, BR-DA, BR-AH, BR-DAH, and PR represent untreated brown rice, ascorbic acid-treated brown rice, degreasing combined with ascorbic acid-treated brown rice, ascorbic acid hydrothermal-treated brown rice, degreasing combined with ascorbic acid hydrothermal-treated brown rice, and polished rice, respectively.

To further investigate the impact of ascorbic acid treatments on the eating quality of brown rice, the sensory evaluation was carried out (Table 2) and Figure 1 illustrated the appearance of cooked brown rice. The bran layer of brown rice hinders the water penetration into the internal grain, leading to a longer cooking time, accompanied by the odor of rice bran [4]. In addition, due to insufficient water uptake, starch in brown rice grain cannot be fully gelatinized during cooking, resulting in hard texture and poor digestibility [9]. And the uneven swelling and gelatinization may cause brown rice grain to easily burst and break, and thus the integrity of cooked brown rice grain is poor [13]. As a result of the above reasons, brown rice (BR) scored the lowest on the sensory scale. However, cooked brown rice treated with ascorbic acid alone improved significantly in terms of grain integrity, stickiness, softness, and hardness, which may be attributed to the enhancement of water uptake and solid loss [21]. Nevertheless, compared with ascorbic acid treatment alone, degreasing combined with ascorbic acid treatment (BR-DA) did not significantly improve the sensory score of cooked brown rice.

Compared with degreasing combined with ascorbic acid treatment (BR-DA), ascorbic acid hydrothermal treatment (BR-AH) further improved the sensory scores (odor, grain integrity and flavor) of cooked brown rice. Furthermore, degreasing combined with ascorbic acid hydrothermal treatment (BR-DAH) markedly improved the sensory scores (odor, stickiness, softness and hardness, flavor and cold texture) of cooked brown rice, and there was no obvious difference between the sensory score items of treated brown rice (BR-DAH) and polished rice (PR) except for odor, although the total sensory score of treated brown rice was still significantly below than polished rice. Hydrothermal treatment can soften the bran layer and promote the volatilization of bran odor of brown rice [9]. Thus, cooked brown rice improved significantly by degreasing combined with ascorbic acid hydrothermal treatment, making it closer to cooked polished rice, thus can markedly improve the consumer acceptability of cooked brown rice.

### 3.3. Effects of Ascorbic Acid Treatments on In Vitro Digestibility of Cooked Brown Rice

**Figure 2:**
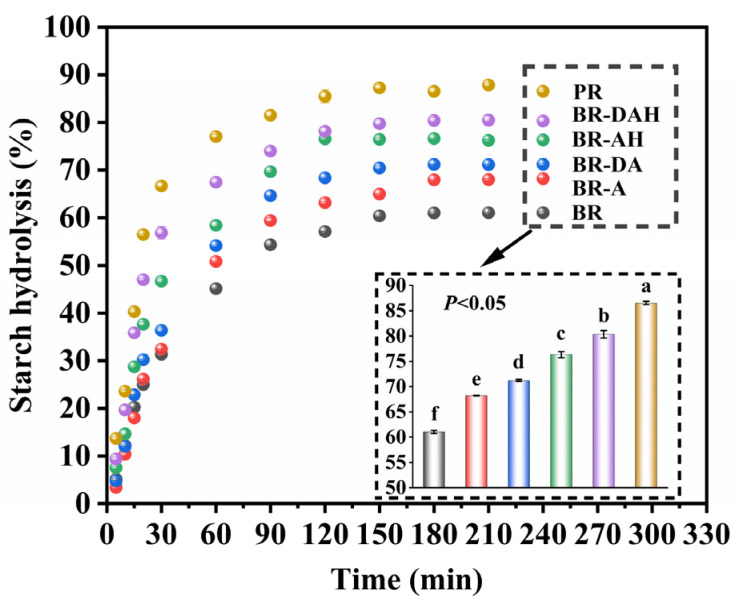
Effects of ascorbic acid treatments on starch hydrolysis of cooked brown rice. BR, BR-A, BR-DA, BR-AH, BR-DAH, and PR represent untreated brown rice, ascorbic acid-treated brown rice, degreasing combined with ascorbic acid-treated brown rice, ascorbic acid hydrothermal-treated brown rice, degreasing combined with ascorbic acid hydrothermal-treated brown rice, and polished rice, respectively. Different letters (a–f) indicate significant differences (*p* < 0.05).

Starch is the main component of brown rice, which is hydrolyzed into glucose in the human body to provide energy, and its digestibility is crucial to human health [36]. For consumer acceptability of brown rice, except for being hard to taste and difficult to cook, another problem is difficult to digest [3,37]. Eating cooked brown rice may cause abdominal pain for some people with poor digestive function [3,37]. Therefore, the improvement of digestibility of cooked brown rice is also very crucial. The effects of ascorbic acid treatments on the digestibility of cooked brown rice were studied, and in vitro digestion curves were shown in Figure 2. Obviously, the starch digestibility of all cooked brown rice samples increased rapidly at the beginning, then gradually reached the maximum value, and finally tended to be stable (Figure 2), indicating that in vitro digestion of starch in cooked brown rice followed the first-order kinetic reaction model [28].

The starch digestibility at the end of the reaction (C_∞_) calculated from the first-order kinetic reaction is listed in Table 3. It has been reported that cooked polished rice grain can swell rapidly and gradually hydrolyze in the process of in vitro digestion, while cooked brown rice grain was difficult for the digestive enzymes to infiltrate, which limits the swelling and hydrolysis of rice grain [38]. In addition, the chemical components of brown rice, such as fiber, lipids, and proteins, potentially wrap starch granules and decrease starch digestibility. Huang et al. [34] showed that the starch digestibility of brown rice also depends on non-starch components, such as fiber, protein, lipids, and polyphenols in the bran. These non-starch components may form a complex with starch, creating a protective layer around the starch, or have an antagonistic effect on digestive enzymes affecting starch digestion. Therefore, the starch digestibility of cooked untreated brown rice was the lowest, while that of cooked polished rice was the highest (Table 3). There were obvious differences in starch digestibility for variously treated samples, and starch digestibility (C_∞_) increased significantly in the order of ascorbic acid treatment alone, degreasing combined with ascorbic acid treatment, ascorbic acid hydrothermal treatment, and degreasing combined with ascorbic acid hydrothermal treatment. Furthermore, the starch digestibility of cooked brown rice could be enhanced from 61.37% of cooked untreated brown rice to 79.53% after degreasing combined with ascorbic acid hydrothermal treatment. This, to a large extent, overcomes the low digestion rate of brown rice. The in vitro digestibility improvement of brown rice by degreasing combined with ascorbic acid hydrothermal treatment is likely to be achieved through increasing contact area with digestive enzymes and through promoting the full swelling and gelatinization of starch [30].

In addition, it can be seen that the starch digestion rate coefficient (k), glycemic index (GI), and hydrolysis index (HI) of brown rice for each treatment were markedly overtopped than those of untreated brown rice (Table 3), and the changing trend was similar to that of starch digestibility (C_∞_), which significantly increased in the order listed in Table 3. This further manifested that degreasing combined with ascorbic acid hydrothermal treatment is the most effective way to improve the digestibility of cooked brown rice. According to the literature reported, the degree of starch gelatinization affects its digestibility [9]. Before cooking, increasing the soaking temperature can help water diffuse into the rice grains, resulting in better starch gelatinization during cooking, thus making the texture of the cooked brown rice softer and hence easy to digest (higher GI value) [9]. In addition, even after treatment, the glycemic index of brown rice is still markedly lower than that of polished rice, and therefore different treatments can be used to obtain mouthfeel-improved brown rice with different GI values suitable for various diabetics. Generally, degreasing combined with ascorbic acid hydrothermal treatment of cooked brown rice decreases hardness and chewiness, increases the stickiness, improves the mouthfeel, and enhances its digestibility.

### 3.4. Effects of Ascorbic Acid Treatments on Cooking Quality of Brown Rice

**Figure 3:**
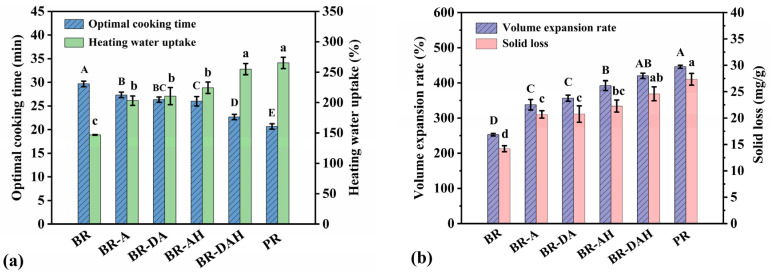
Effects of ascorbic acid treatments on cooking quality of brown rice. Optimal cooking time and heating water uptake (**a**), volume expansion rate and solid loss (**b**). BR, BR-A, BR-DA, BR-AH, BR-DAH, and PR represent untreated brown rice, ascorbic acid-treated brown rice, degreasing combined with ascorbic acid-treated brown rice, ascorbic acid hydrothermal-treated brown rice, degreasing combined with ascorbic acid hydrothermal-treated brown rice, and polished rice, respectively. Different letters (A–E or a–d) indicate significant differences (*p* < 0.05).

The effects of ascorbic acid treatments on cooking quality of brown rice are shown in Figure 3. The water uptake by the inside of brown rice grain is very slow, because the surface layer starch of the rice grain may be gelatinized first due to the priority of water uptake, thus presumably preventing the water from further diffusing into the interior of the grain, making internal starch gelatinization more difficult. Therefore, untreated brown rice had the longest optimal cooking time (OCT). Ascorbic acid treatment alone significantly shortened the OCT of brown rice, meanwhile, ascorbic acid hydrothermal treatment and degreasing combined with ascorbic acid hydrothermal treatment further shortened the OCT of brown rice (Figure 3a). Degreasing combined with ascorbic acid hydrothermal treatment decreased the OCT of brown rice from 29.67 min to 22.67 min. Although the OCT was still significantly higher than polished rice, it was only 2.00 min longer than polished rice, therefore the improvement effect was very significant.

The heating water uptake (HWU), volume expansion rate (VER), and solid loss (SL) were significantly improved by ascorbic acid treatment alone and in combination with degreasing and/or hydrothermal treatment (Figure 3), and the changing trend of the three indexes was similar, showing an increasing trend in accordance with the order of ascorbic acid treatment alone, degreasing combined with ascorbic acid treatment, ascorbic acid hydrothermal treatment, and degreasing combined with ascorbic acid hydrothermal treatment. In particular, the HWU, VER, and SL after degreasing combined with ascorbic acid hydrothermal treatment achieved that of polished rice target. Ascorbic acid can significantly promote SL, thus enhancing the stickiness of brown rice. Sufficient water uptake is necessary for starch gelatinization [39], and the increase in HWU is the direct driving force for the shortening of OCT and the increase in VER and SL. The improvement of cooking quality is directly relevant to the improvement of texture, sensory score, and in vitro digestibility of cooked brown rice.

### 3.5. Effects of Ascorbic Acid Treatments on Relative Crystallinity of Brown Rice

**Figure 4:**
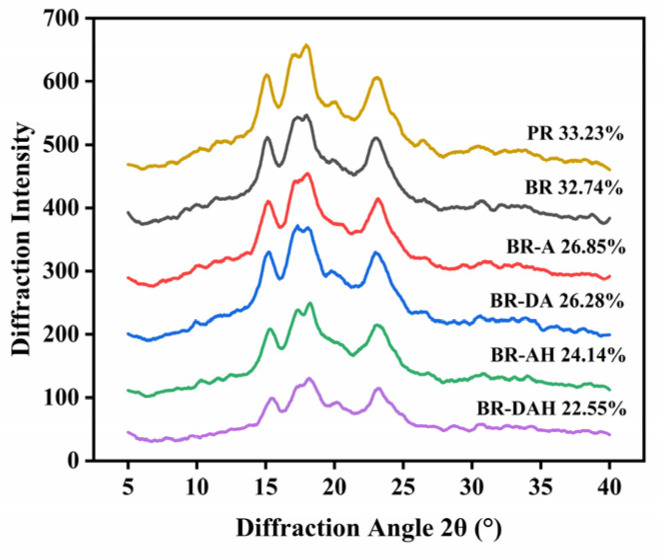
Effects of ascorbic acid treatments on relative crystallinity of brown rice. BR, BR-A, BR-DA, BR-AH, BR-DAH, and PR represent untreated brown rice, ascorbic acid-treated brown rice, degreasing combined with ascorbic acid-treated brown rice, ascorbic acid hydrothermal-treated brown rice, degreasing combined with ascorbic acid hydrothermal-treated brown rice and polished rice, respectively.

The effects of ascorbic acid treatments on relative crystallinity of the treated brown rice are shown in Figure 4. After ascorbic acid treatment alone or in combination, the diffraction peaks at 15°, 17°, 18°, and 23° did not disappear, indicating that the crystalline structure of starch in treated brown rice did not change, which was still typical A-type [40]. Polished rice is retained by removing the bran layer of brown rice, therefore there is almost no difference in the relative crystallinity between the two kinds of untreated rice. The relative crystallinity of untreated brown rice was higher, while the relative crystallinity of brown rice after various treatments with ascorbic acid decreased to varying degrees, and the descending extent increased in turn. The reduction of relative crystallinity showed that the treatments used destroyed the starch crystallites to a certain extent and increased the area of amorphous structure, which was beneficial to water uptake and gelatinization during cooking, but the treatments did not make the crystals disappear or change the crystal type. Park et al. [41] showed that the starch of brown rice after ultrasonic treatment showed an A-type spectrum, and observed that the decreased starch relative crystallinity may be due to the selective degradation of the crystalline region by ultrasonic treatment. Pan et al. [42] found that the starch crystal structure of brown rice was destroyed after cooking, and the crystal structure of starch changed from A-type to amorphous.

### 3.6. Effects of Ascorbic Acid Treatments on Water Droplet Contact Angle of Brown Rice

**Figure 5:**
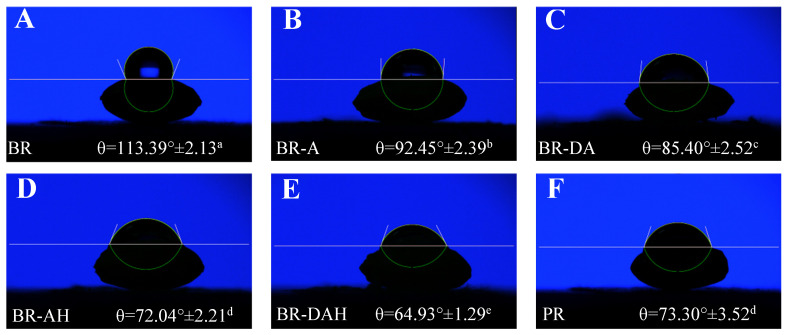
Effects of ascorbic acid treatments on water droplet contact angle of brown rice. The letters of (**A**–**F**) show the water droplet contact angle of BR, BR-A, BR-DA, BR-AH, BR-DAH and PR respectively. BR, BR-A, BR-DA, BR-AH, BR-DAH, and PR represent untreated brown rice, ascorbic acid-treated brown rice, degreasing combined with ascorbic acid-treated brown rice, ascorbic acid hydrothermal-treated brown rice, degreasing combined with ascorbic acid hydrothermal-treated brown rice, and polished rice, respectively. Different letters (a–e) indicate significant differences (*p* < 0.05).

The bran layer of brown rice is a hydrophobic material including lipids, proteins, fibers, and ashes [1]. The effects of ascorbic acid treatments on water droplet contact angle of brown rice grain surface are illustrated in Figure 5. The surface hydrophobicity of brown rice grain was high, and the water droplet contact angle was as high as 113.39°. After being treated with ascorbic acid alone, the water droplet contact angle significantly decreased to 92.45°. Degreasing combined with ascorbic acid treatment, ascorbic acid hydrothermal treatment, and degreasing combined with ascorbic acid hydrothermal treatment further markedly reduced the water droplet contact angle of brown rice grain, and water droplet contact angle of brown rice grain after ascorbic acid hydrothermal treatment reached to that of polished rice. Furthermore, the water droplet contact angle of brown rice grain after degreasing combined with ascorbic acid hydrothermal treatment was far lower than that of polished rice, which indicated that degreasing combined with ascorbic acid hydrothermal treatment significantly improved the surface hydrophilicity of brown rice grain from obvious hydrophobicity to obvious hydrophilicity (contact angle lower than 90°) [43], which was beneficial to the improvement of water uptake of brown rice.

### 3.7. Effects of Ascorbic Acid Treatments on Normal Temperature Water Uptake of Brown Rice

**Figure 6:**
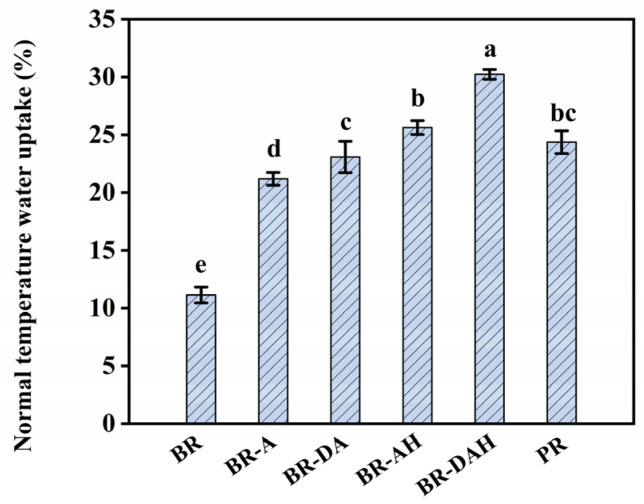
Effects of ascorbic acid treatments on normal temperature water uptake of brown rice. BR, BR-A, BR-DA, BR-AH, BR-DAH, and PR represent untreated brown rice, ascorbic acid-treated brown rice, degreasing combined with ascorbic acid-treated brown rice, ascorbic acid hydrothermal-treated brown rice, degreasing combined with ascorbic acid hydrothermal-treated brown rice, and polished rice, respectively. Different letters (a–e) indicate significant differences (*p* < 0.05).

To further reveal the reason why ascorbic acid improved the eating quality of brown rice, normal temperature water uptake (NTWU) was determined, as presented in Figure 6. Ascorbic acid treatment alone increased the NTWU of brown rice from 11.13% to 21.19%, nearly twice that of untreated brown rice. Degreasing combined with ascorbic acid treatment can further significantly enhance the NTWU of brown rice, and ascorbic acid hydrothermal treatment made the NTWU of brown rice reach the target of polished rice. In particular, degreasing combined with ascorbic acid hydrothermal treatment can increase the NTWU of brown rice to 30.24%, which is markedly higher than that of polished rice. The higher NTWU than polished rice is helpful to the hydration and gelatinization of starch in brown rice, and is the underlying reason for the improvements in texture, mouthfeel, and cooking quality. The increase in NTWU may be attributed to the increase in surface hydrophilicity of brown rice grain. Andrade et al. [44] reported that the reduction of water droplet contact angle of brown rice grain may be related to the water uptake of rice grain. Dobrin et al. [43] reported that the water droplet contact angle of wheat seeds treated by plasma decreased from 92 ± 0.73° to 53 ± 0.85°, which was related to the increase of water uptake by 10–15%. Therefore, the reduction of the water droplet contact angle is believed to be the cause of the increase in NTWU, and the improvement of eating quality and digestibility of brown rice was attributed to the acceleration of water uptake.

### 3.8. Effects of Ascorbic Acid Treatments on Microscopic Morphologies Inside Cooked Brown Rice

**Figure 7:**
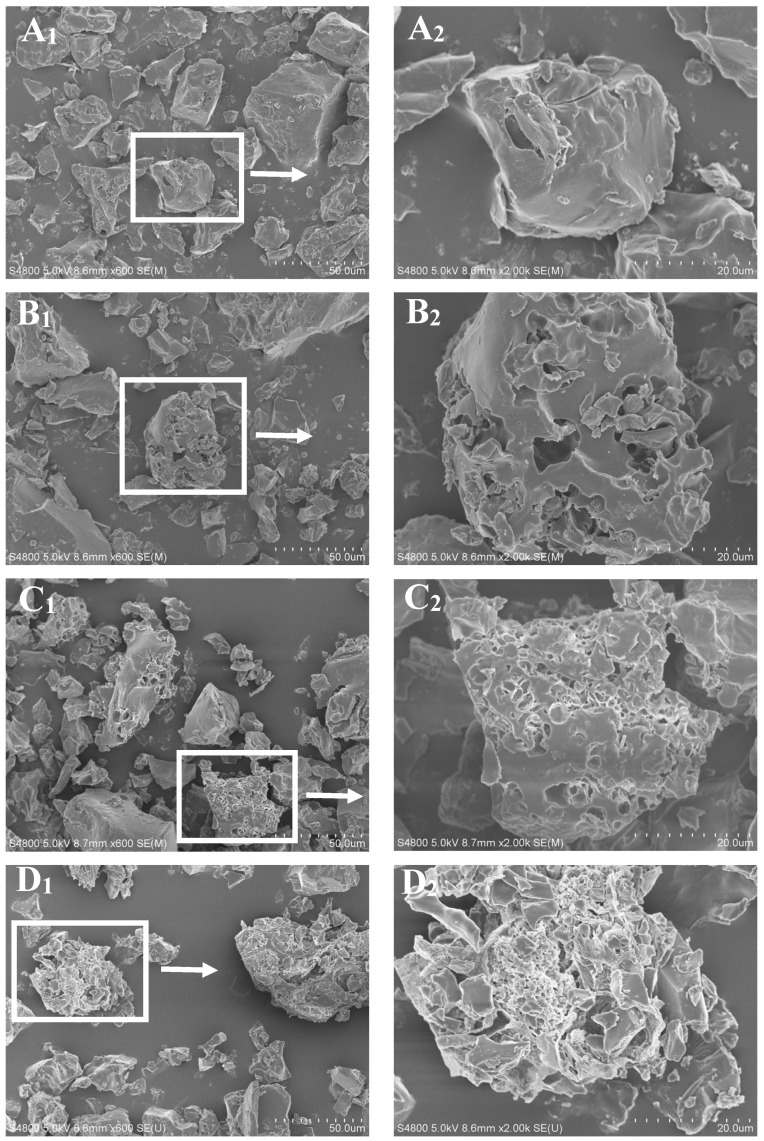

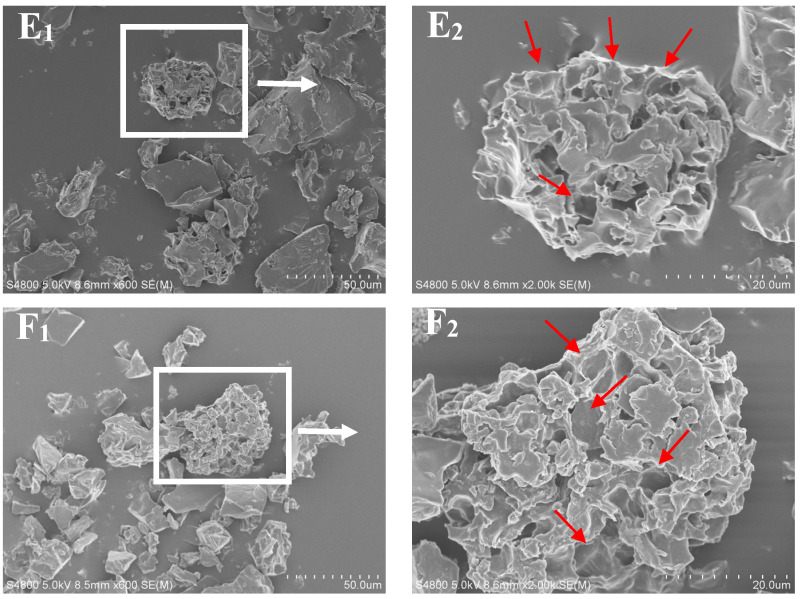
Effects of ascorbic acid treatments on microscopic morphologies inside cooked brown rice. (**A_1_**–**F_1_,A_2_**–**F_2_**) Cooked brown rice of BR, BR-A, BR-DA, BR-AH, BR-DAH, and PR, respectively. BR, BR-A, BR-DA, BR-AH, BR-DAH, and PR represent untreated brown rice, ascorbic acid-treated brown rice, degreasing combined with ascorbic acid-treated brown rice, ascorbic acid hydrothermal-treated brown rice, degreasing combined with ascorbic acid hydrothermal-treated brown rice, and polished rice, respectively. The red arrows in Figure E_2_ and Figure F_2_ show the starch granule abscission pits inside the cooked rice of BR-DAH and PR.

The effects of ascorbic acid treatments on microscopic morphologies inside cooked brown rice are shown in Figure 7. There are many starch granule abscission pits inside the cooked polished rice, and the opening degree of the pits is large (Figure 7F_1_,F_2_), indicating an obvious separation of starch granules [21,23]. Conversely, the interior of cooked brown rice was correspondingly compact and flat, with almost no starch granule abscission pits (Figure 7A_1_,A_2_), which may be due to separation difficulty of starch granules resulting from the low degree of gelatinization. After ascorbic acid treatment alone, some pits appeared inside the cooked brown rice, but the opening degree of the pits was small, indicating that ascorbic acid promoted the gelatinization (Figure 7B_1_,B_2_) of brown rice to some extent. After degreasing combined with ascorbic acid treatment, there were many tiny holes and a few larger pits inside cooked brown rice (Figure 7C_1_,C_2_), both of them were shallow, but the degree of gelatinization appeared to be higher than ascorbic acid treatment alone. After ascorbic acid hydrothermal treatment, the tiny holes inside the cooked brown rice became dense, showing a looser honeycomb structure in the local areas, and more macropores appeared (Figure 7D_1_,D_2_), indicating that ascorbic acid hydrothermal treatment can effectively promote the gelatinization of starch. After degreasing combined with ascorbic acid hydrothermal treatment, there were more starch granule abscission pits inside the cooked brown rice, with a larger opening degree, indicating that starch granules were obviously separated, which is very similar to polished rice (Figure 7E_1_,E_2_). Therefore, degreasing combined with ascorbic acid hydrothermal treatment markedly promoted the separation of starch granules inside cooked brown rice, and improved the uniformity of starch gelatinization, which was consistent with the increases of solids loss and stickiness of the treated cooked brown rice.

In the gelatinization process of the treated brown rice, water is easier to penetrate into the rice grains through the surface layer, which makes the internal starch granules swell adequately and separate each other, therefore improving the degree of starch gelatinization and eating quality of the treated brown rice. The separated starch granules may break more easily, and the small starch molecules can easily migrate outside the rice grain, which helps to improve the stickiness and digestibility of cooked rice. Therefore, the cooked brown rice after degreasing combined with ascorbic acid hydrothermal treatment has higher stickiness, better mouthfeel, and easier digestion. The increase in amorphous structure inside rice grain and more fissures and tiny holes on the surface are consistent with the increase in HWU and SL, showing that the gelatinization degree of brown rice depends on the internal and external permeability of the rice grain.

## 4. Conclusions

For the internal structure of brown rice grains, degreasing combined with ascorbic acid hydrothermal treatment decreased the relative crystallinity of brown rice and thus increased the amorphous structure, which was helpful for water uptake and gelatinization of starch in brown rice. For the surface of brown rice grains, the degreasing combined with ascorbic acid hydrothermal treatment markedly decreased the water droplet contact angle and thus enhanced the surface hydrophilicity. These changes were confirmed by the significant increase in NTWU. Therefore, the increase in internal amorphous structure and surface hydrophilicity enhance the water uptake capacity of brown rice grains in a limited time during cooking, significantly improve the cooking quality, and thus improving the texture, sensory score, and in vitro digestibility of cooked brown rice. The coordination role between interior (relative crystallinity) and surface (contact angle) of rice grain is the underlying reason for the improvement of brown rice eating quality. As for microscopic morphologies of brown rice, degreasing combined with ascorbic acid hydrothermal treatment can contribute to promoting the separation of starch granules inside cooked brown rice grains. The above results show that degreasing combined with ascorbic acid hydrothermal treatment can effectively improve the eating quality and digestibility of brown rice, which is helpful to enhance the acceptability of the prefabricated brown rice after treatment as a staple food consumed.

## Figures and Tables

**Table 1:** Effects of ascorbic acid treatments on texture of cooked brown rice.

Sample	Hardness (N)	Stickiness (N)	S/H Ratio	Chewiness (N)
BR	21.135 ± 1.803 ^a^	0.282 ± 0.077 ^e^	0.013 ± 0.003 ^e^	10.071 ± 1.571 ^a^
BR-A	16.803 ± 1.345 ^b^	0.530 ± 0.089 ^d^	0.032 ± 0.006 ^d^	8.281 ± 0.985 ^b^
BR-DA	16.730 ± 1.094 ^b^	0.630 ± 0.095 ^c^	0.038 ± 0.005 ^c^	8.084 ± 1.120 ^bc^
BR-AH	15.613 ± 1.222 ^c^	0.664 ± 0.098 ^c^	0.043 ± 0.008 ^c^	7.477 ± 1.337 ^c^
BR-DAH	14.421 ± 1.369 ^d^	0.848 ± 0.114 ^b^	0.060 ± 0.011 ^b^	6.461 ± 1.071 ^d^
PR	14.251 ± 1.428 ^d^	1.302 ± 0.233 ^a^	0.092 ± 0.016 ^a^	6.413 ± 1.015 ^d^

Note: BR, BR-A, BR-DA, BR-AH, BR-DAH, and PR represent untreated brown rice, ascorbic acid-treated brown rice, degreasing combined with ascorbic acid-treated brown rice, ascorbic acid hydrothermal-treated brown rice, degreasing combined with ascorbic acid hydrothermal-treated brown rice, and polished rice, respectively. S/H ratio represents the stickiness/hardness ratio. Different letters (a, b, c, d and e) in the same column indicate significant differences (*p* < 0.05).

**Table 2:** Effects of ascorbic acid treatments on sensory score of cooked brown rice.

Sample	Odor (20%)	Appearance and Structure (20%)		Palatability (30%)			Flavor (25%)	Cold Texture (5%)	Sensory Score (100)
		Color and Luster (10%)	Grain Integrity (10%)	Stickiness (10%)	Springiness (10%)	Softness and Hardness (10%)			
BR	14.3 ± 0.7 ^d^	8.0 ± 0.8 ^a^	6.5 ± 0.7 ^c^	6.1 ± 0.6 ^d^	7.3 ± 0.7 ^b^	6.3 ± 0.7 ^d^	17.1 ± 0.7 ^d^	2.6 ± 0.5 ^c^	68.2 ± 1.2 ^e^
BR-A	14.5 ± 0.5 ^d^	8.3 ± 0.8 ^a^	7.3 ± 0.8 ^b^	7.4 ± 0.8 ^c^	7.7 ± 0.8 ^b^	7.0 ± 0.7 ^c^	17.7 ± 0.7 ^cd^	3.0 ± 0.7 ^bc^	72.9 ± 1.7 ^d^
BR-DA	14.8 ± 0.6 ^d^	8.2 ± 0.8 ^a^	7.0 ± 0.8 ^bc^	7.6 ± 0.5 ^bc^	7.4 ± 0.8 ^b^	7.4 ± 0.5 ^bc^	18.2 ± 0.6 ^c^	2.8 ± 0.4 ^c^	73.4 ± 1.1 ^d^
BR-AH	16.0 ± 0.9 ^c^	8.3 ± 0.7 ^a^	8.4 ± 0.5 ^a^	7.9 ± 0.6 ^b^	7.5 ± 0.7 ^b^	7.7 ± 0.5 ^b^	19.6 ± 0.5 ^b^	2.6 ± 0.5 ^c^	78.0 ± 1.6 ^c^
BR-DAH	17.1 ± 0.6 ^b^	8.4 ± 0.7 ^a^	8.6 ± 0.5 ^a^	8.1 ± 0.7 ^a^	8.0 ± 0.8 ^ab^	8.6 ± 0.5 ^a^	21.5 ± 0.8 ^a^	3.4 ± 0.5 ^ab^	83.7 ± 1.6 ^b^
PR	18.0 ± 0.9 ^a^	8.7 ± 0.5 ^a^	8.6 ± 0.7 ^a^	8.2 ± 0.6 ^a^	8.4 ± 0.5 ^a^	8.7 ± 0.5 ^a^	22.1 ± 0.9 ^a^	3.6 ± 0.5 ^a^	86.3 ± 2.3 ^a^

Note: BR, BR-A, BR-DA, BR-AH, BR-DAH, and PR represent untreated brown rice, ascorbic acid-treated brown rice, degreasing combined with ascorbic acid-treated brown rice, ascorbic acid hydrothermal-treated brown rice, degreasing combined with ascorbic acid hydrothermal-treated brown rice and polished rice, respectively. Different letters (a, b, c, d and e) in the same column indicate significant differences (*p* < 0.05).

**Table 3:** Effects of ascorbic acid treatments on starch hydrolysis of cooked brown rice.

Sample	C_∞_ (%)	k × 10^−2^ (min^−1^)	HI (%)	GI
BR	61.37 ± 0.06 ^f^	2.04 ± 0.01 ^f^	60.10 ± 0.06 ^f^	72.70 ± 0.03 ^f^
BR-A	68.86 ± 0.02 ^e^	2.13 ± 0.01 ^e^	68.15 ± 0.10 ^e^	77.12 ± 0.05 ^e^
BR-DA	72.09 ± 0.12 ^d^	2.39 ± 0.01 ^d^	73.17 ± 0.12 ^d^	79.88 ± 0.07 ^d^
BR-AH	76.71 ± 0.18 ^c^	2.88 ± 0.06 ^c^	80.60 ± 0.36 ^c^	83.96 ± 0.20 ^c^
BR-DAH	79.53 ± 0.79 ^b^	3.79 ± 0.03 ^b^	86.93 ± 0.94 ^b^	87.44 ± 0.52 ^b^
PR	86.30 ± 0.42 ^a^	4.35 ± 0.04 ^a^	95.85 ± 0.53 ^a^	92.33 ± 0.29 ^a^

Note: C_∞_, the estimated percentage of starch digestion at the end of reaction; k, the starch digestion rate coefficient; HI, the hydrolysis index; GI, estimated glycemic index. BR, BR-A, BR-DA, BR-AH, BR-DAH, and PR represent untreated brown rice, ascorbic acid-treated brown rice, degreasing combined with ascorbic acid-treated brown rice, ascorbic acid hydrothermal-treated brown rice, degreasing combined with ascorbic acid hydrothermal-treated brown rice and polished rice, respectively. Different letters (a–f) in the same column indicate significant differences (*p* < 0.05).

## Data Availability

Data is contained within the article.

## Supplementary Materials

The following supporting information can be downloaded at: https://www.mdpi.com/article/10.3390/foods12051043/s1, Table S1: Sensory evaluation standard of cooked brown rice.
