# Supplementary material for: Eating Quality and In Vitro Digestibility of Brown Rice Improved by Ascorbic Acid Treatments
Source: Foods. 2023 Mar 1;12(5):1043. doi: 10.3390/foods12051043 (PMC10000754; doi:10.3390/foods12051043)
Supplement: Supplementary file 1 [file foods-12-01043-s001.zip › foods-2195884-supplementary.pdf]

foods

MDPI

Supplementary Material

Eating Quality and In Vitro Digestibility of Brown Rice Improved by Ascorbic Acid Treatments

Qin Wei [1], Yubao Guo [1,*], Kang Tu [2], Xiuling Zhu [1], Dan Xie [1] and Xinyu Liu [1]

[1] School of Biological and Food Engineering, Anhui Polytechnic University, Wuhu 241000, China
[2] College of Food Science and Technology, Nanjing Agricultural University, Nanjing 210095, China
* Correspondence: gyb346@ahpu.edu.cn; Tel.: +86-553-2871-254

Table S1. Sensory evaluation standard of cooked brown rice.

Primary index score	Secondary index score	Evaluation criteria	Score
Odor (20)	Purity and richness (20)	Unique aroma of rice, aroma	18~20
		Unique aroma of rice, fragrance	15~17
		Unique aroma of rice, slight aroma	12~14
		No fragrance, bran smell	7~12
		Unpleasant smell	0~6
Appearance (20)	Color and luster (10)	Color close to raw grain, luster	8~10
		Slightly dark color, less gloss	6~7
		Obviously different from raw grain	0~5
	Grain integrity (10)	Compact structure, good integrity	8~10
		Part of compact structure or good integrity	6~7
		Broken rice grain	0~5
Palatability (30)	Adhesiveness (10)	Sticky, not easy to stick to the teeth	8~10
		Sticky, not to stick to the teeth	6~7
		Sticky teeth, or non-sticky	0~5
	Springiness (10)	Moderate chewiness	8~10
		Slightly Chewy	6~7
		Loose and hard, with slag	0~5
	Hardness (10)	Moderate hardness and softness	8~10
		Slightly hard or soft	6~7
		Hard or soft	0~5
Flavor (25)	Purity and richness (25)	Rich fragrance, sweetness	22~25
		Light fragrance, sweetness	18~21
		No fragrance and sweetness, no Peculiar smell	16~17
		No fragrance and sweetness, peculiar smell	0~15
Cold texture (5)	Agglomeration, viscoelasticity, hardness (5)	Relatively loose, with good viscoelasticity and moderate hardness	4~5
		Agglomerate, poor viscoelasticity, slightly hardened	2~3
		Hardening, poor viscoelasticity, slightly hard	0~1
